# The effect of different cooking methods on sensory attributes, physicochemical properties, and microbial safety of ostrich meat (*Struthio camelus*)

**DOI:** 10.5455/javar.2024.k765

**Published:** 2024-03-31

**Authors:** Nermeen Makram Louis Malak

**Affiliations:** Department of Food Hygiene and Control, Faculty of Veterinary Medicine, Cairo University, Cairo, Giza, Egypt

**Keywords:** Heat treatments, ostrich meat, physicochemical properties, sensory attributes, texture evaluation

## Abstract

**Objective:**

The present work aimed to determine the impact of various cooking methods on sensory attributes, microbial safety, and physicochemical characteristics of ostrich meat to specify the best cooking method that guarantees the microbial safety of the meat as well as maintains nutritional values and is highly attractive to consumers.

**Materials and Methods:**

One hundred fresh leg muscles of ostriches were divided into five groups. Different cooking techniques were used for each group, as follows: roasting, boiling, grilling, frying, and microwaving. Each method was examined by evaluating the impact of various cooking methods on sensory attributes, proximate chemical analysis, protein and fat oxidation parameters, microbial load, changes in color, and the shear force of ostrich meat.

**Results:**

The oven-roasting and grilling methods are highly recommended and more appealing to consumers since they produce tenderer and juicier meat, cause less cooking loss (CL), and maintain the nutritive value of ostrich meat; however, they have the highest protein and fat oxidation rates. On the other hand, boiling and frying methods revealed good fat oxidation parameters, the highest CL, and preserved nutritional value, but unfortunately, they were not highly preferred by consumers. From a hygienic point of view, grilling and microwave cooking are the methods that ensure the microbiological safety of cooked ostrich meat, as they significantly reduce Enterobacteriaceae and psychrotrophic bacterial counts, among other cooking methods.

**Conclusion:**

The oven roasting and grilling methods were the most preferable thermal cooking techniques, as they achieved the highest acceptability to consumers and maintained the nutritive values of ostrich meat.

## Introduction

Ostriches (*Struthio camelius*) are flightless birds (ratites). The ostrich was originally found in Syria, Saudi Arabia, and Africa, but now it is only present in Africa. Ostrich farming began in South Africa as a means of producing leather and later textiles [1]. Nevertheless, ostriches are grown for their meat all over the world. This industry is growing, and its uniqueness is one of its attractive properties. In addition to consumers, geneticists, farmers, and animal production researchers are all fascinated by this meat. Ostrich production will focus on providing ostrich meat as an alternative to traditional meats in the future. There are about 12,000 to 15,000 tons of ostrich meat produced worldwide. Steaks, burgers, sausages, and fresh meat are among the main ostrich meat products. South Africa produces approximately 60% of the total production of ostrich meat, while the remaining 40% is sold to other nations worldwide, including Australia, Poland, the United States, Spain, and Middle Eastern nations [2].

Ostrich meat is thought to be the most nutritious and healthy red meat. It has a flavor resembling beef. In addition, it is preferred by people suffering from hypertension, obesity, and anemia since it has a higher iron, manganese, and phosphorous content, as well as a lower sodium content than other meats. Due to its high polyunsaturated fatty acid content, it is better than beef and other red meats [3]. Interestingly, it is ideal for consumers aiming to control their weight since it contains less fat compared to turkey, chicken, and beef. Furthermore, ostrich meat has a low fat-to-protein ratio, while turkeys and cattle have higher fat-to-protein ratios. In addition, compared to beef and turkey, it has less cholesterol [4,5].

Cooking meat is considered an important procedure as it reduces the microbial load, enhances the taste, improves nutrient digestion, and softens the texture. Moreover, cooking meat at different temperatures may affect the solubility of meat proteins, water-holding capacity, cooking loss (CL), meat shrinkage, juiciness, and tenderness [6]. In fact, ostrich meat is tougher than beef, as it contains less connective tissue and has a lower collagen content than beef. Furthermore, it contains low fat levels. On the other hand, if ostrich meat is cooked for a long time at a high temperature, it may result in a dry sensation. Consequently, it should be cooked in a way that achieves its juiciness and tenderness as well as preserves its nutritional value and sensory properties. The tenderness of ostrich meat is greatly dependent on several factors, such as bird age, chemical composition, cooking methods, cooking temperatures, and the anatomical location of muscle [7].

Worldwide, the most popular methods used by consumers for thermal cooking of different types of meat are pan-frying, boiling, microwaving, grilling, and oven-roasting [8]. These techniques result in several changes in the color of meat, protein digestibility, oxidation of fat, and sensory changes in meat characteristics [9]. In these regards, there were numerous studies that studied the impact of various cooking techniques on beef, pork, fish, chicken, goose, and rabbit meat [10–14]. In addition, several studies claimed that there was a lack of supporting data studying the characteristics of ostrich meat for incorporation in ostrich meat products. To the best of our knowledge, no research has been presented on the effects of cooking temperatures on appearance, color, texture, juiciness, tenderness, nutritional quality, changes in fat and protein breakdown, or the microbial safety of ostrich meat. Consequently, the purpose of the study was to assess the effects of the most popular cooking methods on the microbial safety, physicochemical characteristics, and sensory characteristics of ostrich meat to determine the most healthy, nutritious, and safe way to cook ostrich meat.

## Materials and Methods

### Ethical approval

The faculty of veterinary medicine at Cairo University in Giza, Egypt, gave its approval to the research plan. It was going along with the recommendations and regulations of the animal welfare and ethics committee. Ostrich meat samples were obtained from local markets without using living animals.

### Samples of meat

Hundreds of fresh leg muscle (*M. ilifibularis*) samples (without bone and skin) were obtained from both female and male ostriches (*Struthio camelus*), which were slaughtered at the commercial ostrich slaughterhouse in Cairo, Egypt. The ostriches were about 60–70 kg and 2–3 years old. The ostrich meat samples were taken six hours after the animal was slaughtered and transported in a refrigerated ice box for further processing and examination. The meat samples were chunked into cubes (2 × 2 × 1 cm) and maintained at 4°C for about 24 h in the refrigerator.

### Heat treatments

Five groups of 20 samples each were formed from the 100 samples of ostrich meat. Each group tested a variety of cooking techniques. The first, second, third, fourth, and fifth groups were prepared individually by roasting in the oven, boiling, frying, grilling, and microwave cooking, respectively. The experiment was repeated three more times on distinct days. The first group was roasted for 30 min at 150°C in a roasting oven covered with aluminum foil. The second group was boiled in separate plastic bags for 30 min at 100°C in a cooking vessel. In a frying pan at 150°C, the samples in the third group were fried, and every 3 min were turned over until thoroughly cooked for 20 min. The fourth group was grilled for around 20 min total, flipping over every 3 min, on an electric grill set to 150°C. The fifth group was prepared in a microwave for 6 min at 1,200 W. No additives were added to ostrich meat samples during different cooking procedures so that we could analyze just the changes brought on by the various cooking techniques. Ostrich meat samples were heat treated at their core temperature (75°C), which is considered a safe temperature for consumption of the ostrich meat. Hand-held thermometers calibrated previously were used to monitor the temperature in the core of ostrich meat samples. The different cooking groups were exposed to different investigations.

### Approximate composition analyses

Cooked ostrich meat samples were measured for moisture, fat, protein, and ash (gm/100 gm) following the Association of Official Analytical Chemists (AOAC) method [15].

### Measurement of shelf life indicators (deterioration criteria)

The pH values of the samples were assessed after mixing 5 gm of cooked samples with distilled water and measured using a pH meter as per the procedure outlined by Kandeepan et al. [16]. The total volatile basic nitrogen (TVBN) was determined according to the steps given by Kearsley et al. [17]. In addition, the Du and Ahn technique [18] was used to calculate the quantity of thiobarbituric acid (TBA) per kilogram of meat, which was then expressed in milligrams of malonaldehyde (MDA) (mg/kg MDA).

### Determination of free fatty acid (FFA) content and acid number (AN)

Cooked samples were examined for their FFA content using the established methodology developed by American Oil Chemists Society (AOCS) [19].

### Evaluation of the color of different heat-treated ostrich meat samples

The color of all cooked samples was assessed following the technique mentioned by Shin et al. [20] by Konica Minolta Chroma Meter (CR-400, Japan).

### Determination of the shear force (SF) of different heat-treated ostrich meat samples

According to Shackelford et al. [21], four samples (1 × 1 × 1 cm) of each cooked ostrich meat sample were cut in the longitudinal axis of muscle fibers, and the SF of each sample was calculated with a Warner Bratzler shear apparatus.

### Measurement of cooking loss (CL) of different heat-treated ostrich meat samples

According to Woloszyn et al. [22], by comparing the weight difference between raw and prepared samples to the weight of the raw sample, we can determine the percentage of CL.

### Bacteriological examination of different heat-treated ostrich meat samples

Samples were serially ten-fold diluted. The plate count agar can be used to culture both mesophilic bacteria and was incubated for 48 h at 36°C [23], while psychrotrophic bacteria were cultivated for 10 days at 7°C [24]. In addition, enterobacteriaceae were counted after incubating at 37°C for 24 h on inoculated Violet-Red-Bile Glucose agar [25].

### Sensory analysis

The ostrich meat samples that were prepared by different methods of thermal treatments were evaluated in accordance with the recommendations made by Meilgaard et al. [26]. 35 qualified panelists evaluated the flavor, appearance, color, juiciness, tenderness, and general acceptability in a random sequence. Then they give a single number ranging from 1 for severely undesirable samples to 9 for highly preferred samples.

### Statistical analysis

The results of all measurements were statistically analyzed with SPSS Statistics 27.0 (mean values ± SE). All means of the five treatments for the variables were compared using ANOVA analysis. In addition, the *Post-Hoc* hoc (least squares difference) approach was applied to make a comparison between all of the parameter means again. A difference was deemed significant when it met the (*p* < 0.05) cut-off.

## Results and Discussion

### Changes in the chemical composition of ostrich meat cooked by different cooking methods

Generally, cooking ostrich meat with the five cooking techniques resulted in a reduction of the water content and increased ash, fat, and protein contents (Table 1). The moisture content values revealed that oven-roasted and grilled ostrich meat samples exhibited the highest moisture levels as well as decreasing amounts of ash, fat, and protein. Conversely, boiling as well as frying ostrich meat significantly reduced moisture content, accompanied by an increase in fat, protein, and ash. However, microwave cooking caused intermediate effects on fat, moisture, ash, and protein levels. The results may be attributed to the fact that different methods of thermal cooking lead to the denaturation of proteins, loss of water from the meat surface, muscle fiber shrinkage, and a decrease in the ability of meat to hold water. Consequentially, the concentration of different muscle constituents increases. Similarly, prolonging the time and increasing the temperature of boiling for the cooking of the emu meat (*Dromaius novaehollandiae*) significantly (*p* < 0.05) decreased moisture (74.49/100 gm) in raw meat versus 68.11/100 gm in cooked meat) and protein (24.39/100 gm in raw meat *vs.* 17.07/100 gm in cooked meat) [27].

### Changes in the microbial load of ostrich meat cooked by different methods

Changes in microbial count in ostrich meat subjected to five cooking procedures are revealed in Table 2. The findings from this study demonstrated that aerobic plate counts (APC) of ostrich meat samples cooked in the oven, boiling, frying, grilling, and microwave did not differ significantly. On the other hand, when ostrich meat samples were microwaved, the psychrotrophic bacterial count significantly decreased, and when the samples were grilled, it decreased below the detectable limits (>2.00 log_10_ CFU/gm). However, psychrotrophic bacterial counts of oven-roasted, boiled, and fried samples did not significantly differ except for samples cooked by oven-roasting, where Enterobacteriaceae reached 1.51 log_10_ CFU/gm. All samples of prepared ostrich meat samples were below the detectable limit. Thus, the microwave and grilling methods are the easiest to apply, and therefore they are the best ways to ensure the microbiological safety of cooked ostrich meat. The results could be due to the fatal impact of microwaves on the survival of microorganisms [28]. The findings agreed with those obtained by Yilmaz et al. [29], who stated that cooking meatballs by microwave decreased the number of microorganisms by 3–4 logs, as well, cooking these meatballs by oven roasting and grilling reduced the microbial count by 2–3 log cycles. Furthermore, GÖK et al. [30] found that oven-cooked beef had significantly lower APC and coliform counts as well as significantly higher psychrotrophic counts as compared to sous-vide-cooked samples.

### Changes in the fat oxidation and protein deterioration parameters of ostrich meat subjected to various cooking methods

In general, cooking meat raises the pH because it separates peptide chains, alters acid group electric charges, and produces new alkaline compounds [31]. In regards to cooking techniques, the results showed that boiling ostrich meat samples revealed the highest pH values (Table 3), whereas other cooking techniques did not result in a considerable change in the pH values. Similarly, Mohamed et al. [32] stated that boiling beef increased pH more than samples prepared by roasting as well as frying. In addition, Nithyalakshmi and Preetha [27] revealed that the pH values of emu meat (*D. novaehollandiae*) increased by extending the time and temperature of boiling.

TVBN values increased significantly in ostrich meat samples prepared by frying, microwaving, and boiling than in samples prepared by roasting (Table 3). Moreover, the allowed limit (20 mg/100 g) for ostrich meat as specified by the Egyptian Standard Specification (E.S.) [33] was not exceeded by TVBN levels of samples cooked using various techniques. The findings were consistent with TVBN measurements made by Abdel-Naeem et al. [13], who reported that when compared to TVBN values of samples prepared by roasting, microwaving, and grilling (10.08, 9.52, and 9.66 mg/100 gm), respectively, rabbit meat that was cooked with frying and boiling revealed higher TVBN values of 11.76 and 11.20 mg/100 gm.

The oxidation of fat during cooking has a significant impact on the sensory qualities of ostrich meat as well as on human health. FFA, TBA, and AN are reliable fat oxidation indicators of the extent of lipid breakdown brought on by heat treatment [34]. The results (Table 3) revealed that the TBA, FFA, and AN values of oven-roasted and microwave-cooked ostrich meat samples were significantly high, although there were insignificant changes between these values in samples prepared by boiling, frying, and grilling. However, none of the TBA values from samples cooked using various techniques were above the ostrich meat permissible limits (0.9 mg MDA/kg) established by the E.S. [33]. In this regard, Brenes et al. [35] explained that increasing the rate of fat oxidation parameters in microwave-cooked samples increases oil thermal oxidation. This is equivalent to the fat oxidation caused by frying in oil for four hours. Microwave energy produces single oxygen, which accelerates the beginning of the fat oxidation process more than normal oxygen. In addition, Weber et al. [36] confirmed that microwave-cooked catfish fillets caused greater fat oxidation than roasted samples. Furthermore, Abdel-Naeem et al. [13] revealed that preparing rabbit meat in the microwave significantly increased TBA, FFA, and AN values more than samples prepared by boiling, grilling, pan-frying, and oven-roasting.

**Table 1.** Chemical composition (gm/100 gm) of ostrich meat subjected to various cooking methods.

	Oven	Boiling	Frying	Grilling	Microwave
Moisture	74.37^a^ ± 0.07	67.89^c^ ± 0.37	67.78^c^ ± 0.08	74.33^a^ ± 0.59	70.84^b^ ± 0.52
Fat	2.27^b^ ± 0.05	3.45^a^ ± 0.32	3.38^a^ ± 0.10	2.31^b^ ± 0.19	2.43^b^ ± 0.08
Protein	22.03^c^ ± 0.46	26.23^a^ ± 0.32	26.40^a^ ± 0.30	22.06^c^ ± 0.24	25.20^b^ ± 0.14
Ash	1.13^b^ ± 0.50	2.07^a^ ± 0.11	2.35^a^ ± 0.10	1.15^b^ ± 0.18	1.39^b^ ± 0.24

^a–c^ means with different superscripts within the same row for each parameter are significantly (*p* <0.05) different.

Values represent the means ±SE, *n* = 20 samples in each group.

**Table 2.** Microbial count (Log_10_ CFU/gm) (mean ± SE) of ostrich meat exposed to different cooking methods.

	Oven	Boiling	Frying	Grilling	Microwave
APC	2.86^a^ ± 0.03	2.83^a^ ± 0.06	2.93^a^ ± 0.02	2.69^a^ ± 0.49	2.79^a^ ± 0.14
Psychrotrophs	1.75^a^ ± 0.39	1.62^a^ ± 0.22	1.51^a^ ± 0.81	<2.0^b^ ± 0.0	0.87^b^ ± 0.87
Enterobacteriaceae	1.51^a^ ± 0.59	<2.0^b^ ± 0.00	<2.0^b^ ± 0.0	<2.0^b^ ± 0.0	<2.0^b^ ± 0.0

APC = aerobic plate count.

^a–b^ means with different superscripts within the same row for each parameter are significantly (*p* < 0.05) different. Values represent the means ±SE, *n* = 20 samples in each group.

### CL, color evaluation, and SF changes in ostrich meat subjected to various cooking methods

The cooking process results in a loss of both soluble and liquid matter. As the temperature increases, the water content decreases, and fats and proteins increase. As a result, most of the CL was water due to the coagulation and denaturation of proteins, thus reducing water trapped within protein structures when preparing meat [37]. Consequently, the percentage of CL is strongly correlated with the moisture content mentioned in Table 1. In this table, samples cooked by frying and boiling achieved the least amount of water and the highest ash, protein, and fat levels. The highest percentage (%) of CL is also found in ostrich meat prepared by boiling and frying, followed by microwave-cooked samples. Interestingly, oven-roasted and grilled ostrich meat have the highest moisture content along with the lowest CL percentage (Table 4). The results were similar to those informed by Dal Bosco et al. [31], who published that pan-fried rabbit meat samples achieved a higher CL (36.49%) than boiled and roasted samples (31.05% and 30.22%, respectively). Furthermore, Juárez et al. [37] stated that the greatest moisture loss percentage was measured in buffalo meat cooked by frying and grilling. However, boiling caused low moisture loss because water entered throughout the cooking process. In addition, Rasinska et al. [38] observed high CL in oven-roasted rabbit meat compared with boiled samples.

Cooking ostrich meat generally causes the water content to decrease, leading to muscle fibers opening and scattering light, thus increasing the lightness values after cooking all samples [38]. In addition, the differences in redness and yellowness values in samples prepared using various cooking techniques may be related to the differences in myoglobin denaturation degree produced by each technique. Table 4 presents the color evaluation of ostrich meat broiled by different cooking techniques. The finding revealed that oven-roasted and grilled samples had the lowest L* values, accompanied by a subsequent significant increase in a* and b* values. In contrast, the boiled and microwaved groups had the highest significant L* values, with subsequent decreasing values of a* and b*. In addition, fried ostrich meat achieved moderate L*, a*, and b* values. Consequently, oven-roasted and grilled meat’s yellowish-red color is more attractive to consumers than whiter, boiled, and microwaved ostrich meat. This is because the change from the attractive red color of oxymyoglobin to the less acceptable brown color of metmyoglobin lowers a* values and eventually causes the meat to be unpleasant to consumers. Among the various cooking methods, oven-roasted, grilled, and fried samples achieved the most attractive appearance to consumers due to low lightness (L*) values and high values of yellowness (b*) and redness (a*). The findings agreed with those stated by Abdel-Naeem et al. [13], who noticed a significant increase in L* values and a decrease a* and b* values in broiled rabbit meat with microwave and boiling. While oven-roasted and grilled samples achieved the lowest L* value and the highest b* and a* values, which are more attractive to consumers, increasing the cooking temperature of the boiled meat of emu (*D. novaehollandiae*) significantly improved L* and b* values and reduced values of a* [27]. Similar findings were made by Zhang et al. [39], who revealed higher L* and lower b* and a* values in boiled rabbit meat than in oven-roasted and fried samples.

**Table 3.** Protein deterioration and fat oxidation parameters (mean ± SE) of ostrich meat exposed to different cooking methods.

	Oven	Boiling	Frying	Grilling	Microwave
pH	6.57^b^ ± 0.18	7.46^a^ ± 0.15	6.44^b^ ± 0.09	6.64^b^ ± 0.14	6.73^b^ ± 0.07
TVBN	4.29^bc^ ± 0.09	5.46^a^ ± 0.08	4.76^ac^ ± 0.29	5.51^a^ ± 0.33	5.60^a^ ± 0.40
TBA	0.81^a^ ± 0.17	0.48^b^ ± 0.00	0.72^b^ ± 0.09	0.64^b^ ± 0.09	0.84^a^ ± 0.03
FFA	0.18^a^ ± 0.00	0.13^b^ ± 0.01	0.13^b^ ± 0.01	0.14^b^ ± 0.00	0.19^a^ ± 0.00
AN	0.36^a^ ± 0.00	0.26^b^ ± 0.03	0.27^b^ ± 0.03	0.28^b^ ± 0.01	0.38^a^ ± 0.00

TVBN = total volatile base nitrogen (mg%); TBA = thiobarbituric acid (mg malonaldehyde/kg); FFA = free fatty acid % as Oleic acid; AN = acid number (mg NaOH/gm).

^a–d^ Means with different superscripts within the same row for each parameter are significantly (*p* < 0.05) different. Values represent the means ±SE, *n* = 20 samples in each group.

**Table 4.** CL, color evaluation, SF parameters (mean ± SE) of ostrich meat exposed to different cooking methods.

Parameters		Oven	Boiling	Frying	Grilling	Microwave
Cooking loss	CL	23.46^c^ ± 0.40	38.64^a^ ± 0.48	38.68^a^ ± 0.51	23.30^c^ ± 0. 68	34.27^b^ ± 0.40
Color evaluation	L*	41.64^e^ ± 0.51	49.21^b^ ± 0.16	46.10^c^ ± 0.04	43.65^d^ ± 0. 14	50.00^a^ ± 0.01
	a*	12.59^a^ ± 0.10	6.70^d^ ± 0.72	8.74^c^ ± 0.11	9.02^b^ ± 0.22	5.51^e^ ± 0.30
	b*	8.04^a^ ± 0.13	5.87^c^ ± 0.16	7.12^b^ ± 0.05	8.62^a^ ± 0.23	3.67^d^ ± 0.28
	SF(N)	2.06^c^ ± 0.18	6.09^a^ ± 0.61	5.30^a^ ± 0.07	2.18^c^ ± 0.44	4.04^b^ ± 0.30

CL = cooking loss (%). L* = lightness; a* = redness; b* = yellowness; SF = shear force (N).

^a–e^means with different superscripts within the same row for each parameter are significantly (*p* < 0.05) different. Values represent the means ±SE, *n* = 20 samples in each group.

The SF is considered one of the key factors that determines meat tenderness. Results exhibited that oven-roasting and grilling methods resulted in the lowest significant (*p* < 0.05) SF (Table 4). While boiled and fried samples showed the highest significant SF, followed by microwave-cooked samples. Consequently, oven-roasted and grilled ostrich meat is more palatable and tender to consumers than samples cooked by other methods. Similarly, Abdel-Naeem et al. [13] found that boiled and fried rabbit meat samples increased SF values. while microwave, oven-roasting, and grilling caused the lowest SF values. Furthermore, Dal Bosco et al. [31] noticed that the SF values of pan-fried (49.43 N) rabbit meat samples were higher than the values of roasted and boiled samples (45.60 and 35.40 N, respectively). However, Fabre et al. [40] reported that oven cooking produced highly significant SF values in different muscles of steers in comparison with griddle plates and water bath-cooked beef steaks.

### Changes in sensory attributes of prepared ostrich meat with different methods of cooking

**Figure 1.**
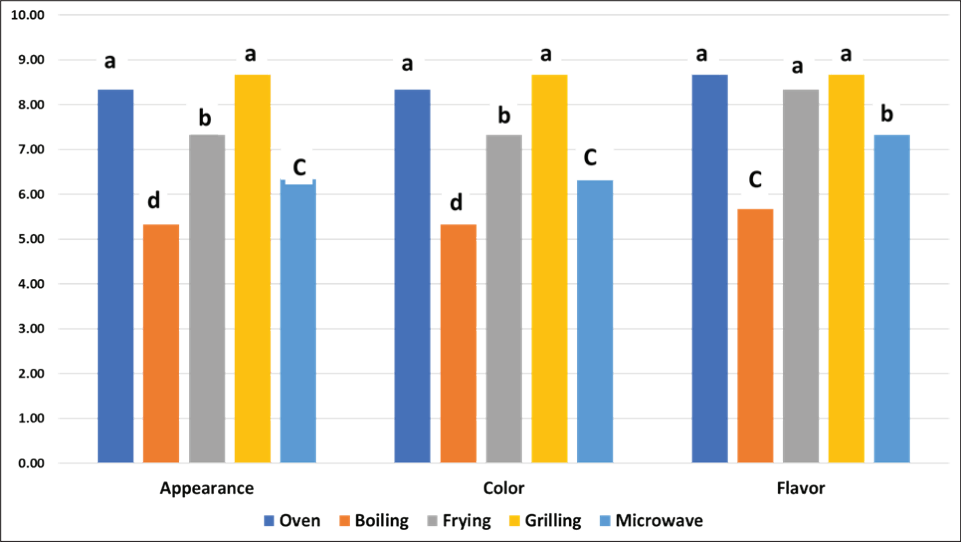
Changes in appearance, color, and flavor of ostrich meat subjected to different cooking methods.

As shown in Figure 1, ostrich meat subjected to various cooking methods changed its sensory attributes (color, appearance, and flavor). In terms of appearance and color, boiling or microwave-cooking ostrich meat resulted in the lowest scores. In contrast, oven-roasted and grilled samples showed the most attractive appearance and color. Moreover, grilling, frying, and oven roasting produced the highest and most appealing flavor. The appearance and color panel scores of fried ostrich meat were higher than those of those that had been boiled or microwaved.

**Figure 2.**
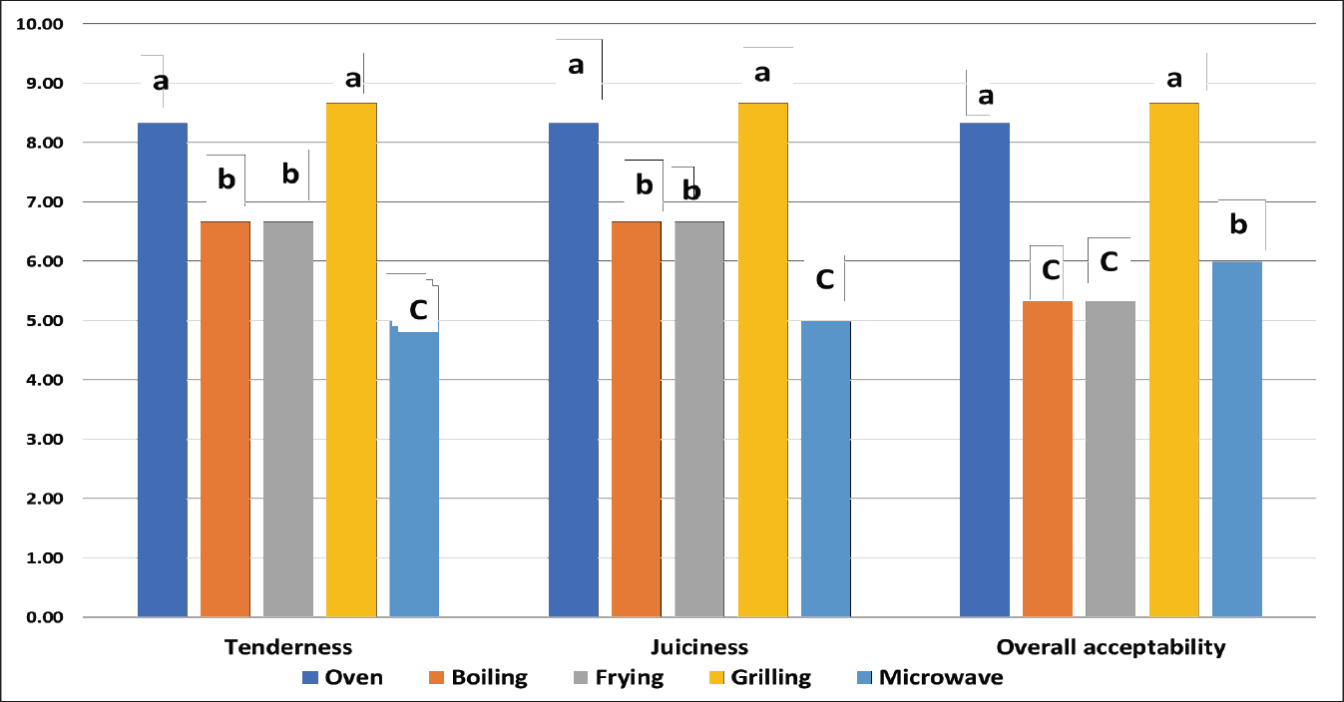
Changes in tenderness, juiciness, and overall acceptability of ostrich meat subjected to different cooking methods.

Concerning other cooking methods, oven-roasted and grilled samples produced the most pleasant, juicy, and tender meat (Fig. 2). Meanwhile, grilled and fried samples were more tender and juicier than microwave-cooked samples. As a result, the panelists found the grilled and oven-roasted samples to be the most acceptable. Microwave-cooked meat samples were slightly more acceptable than fried and grilled meat samples, likely due to their better flavor. While boiled and microwaved samples exhibited the lowest overall acceptability scores, Similar findings indicated that oven-roasted and grilled rabbit meat achieved high panelist scores for tenderness, juiciness, and overall acceptability, while microwaving and boiling yielded significant decreases in the panelists’ scores for flavor, appearance, color, and general acceptability [13]. In addition, oven-roasting of beef improved the juiciness, flavor, color, and overall acceptability scores more than sous-vide samples [30]. Moreover, preparing chicken sticks in the oven and microwave resulted in the highest juiciness, tenderness, and overall acceptability, while samples prepared by grilling achieved the best flavor, color, and overall acceptability scores compared with boiled chicken sticks [41].

According to the sensory evaluations obtained, panelists prefer oven-roasted and grilled ostrich meat due to their attractive flavor, appearance, color, tenderness, juiciness, and overall acceptability (Figs. 1 and 2). Moreover, based on the measurement of texture (SF) and color, ostrich meat samples cooked in oven and grilling were more palatable and attractive to consumers due to their more yellowish–red color as well as being more tender and juicier than the whiter and tougher texture of meat cooked by boiling, frying, and microwave (Table 4). Moreover, roasting and grilling maintained the meat’s nutritional value (Table 1) and achieved the lowest CL (Table 4). From a hygienic point of view, grilling and microwave cooking are the methods that ensure the microbiological safety of cooked ostrich meat, as they significantly reduce the Enterobacteriaceae and psychrotrophic bacterial counts among other cooking methods (Table 2).

## Conclusion

From the obtained results, oven-roasting and grilling cooking methods are highly recommended and more attractive to consumers since they induce a desirable appearance, are more tender and juicier, cause less CL, and maintain the nutritive value of ostrich meat; nevertheless, they have the highest protein and fat oxidation parameters. On the other hand, boiling and frying methods revealed good fat oxidation parameters, the highest CL, and preserved the nutritional value, but unfortunately, they were not highly preferred by consumers due to their plain color, being tougher and less juiciness, as well as high protein oxidation parameters. Moreover, microwave cooking revealed high protein and fat oxidation parameters, more toughness, high CL, less juiciness, unacceptable color and flavor, and overall acceptability. From a hygienic point of view, grilling and microwave cooking are the methods that ensure the microbiological safety of cooked ostrich meat, as they significantly reduce Enterobacteriaceae and psychrotrophic bacterial counts, among other cooking methods.

## List of Abbreviations

a*, redness; AN, acid number; APC, aerobic plate count; b*, yellowness; CL, cooking loss; FFA, free fatty acid; SF, shear force (N): TVB-N, total volatile base nitrogen; TBA, thiobarbituric acid; L*, lightness.
